# Literary Notes

**Published:** 1908-01

**Authors:** 


### LITERARY NOTES.

P. Blakiston's Son Company, Philadelphia, announce a new fifth edition of Jacobson's Operations of Surgery, thoroughly revised, containing an increase of 583 pages and 227 illustrations over the last, which makes it practically a new book in many particulars. Price: cloth, $12; half morocco, $14. Further detailed information will be sent upon application to the publishers.

W. B. Saunders Company, medical publishers of Philadelphia and London, announce that they have found it necessary to issue another revised edition of their illustrated catalogue of medical and surgical books. In looking through the copy we have received, we find that since the issuance of the last edition six months ago, the publishers have placed on the market some twenty-five new books and new editions—truly an indication of publishing activity. The colored insert plate from Keen's new surgery, which enhanced the value of the former edition, has been replaced by a new one from the second volume of the same work, and this alone gives the catalogue a real value. A copy will be sent to any physician upon request.

The Anglo-American Medical Association, in Berlin, has issued its annual report for the year 1907, which contains much of interest for American physicians. Besides a list of officers and members it gives the dates of meetings and other general items that English speaking physicians, visiting Berlin, will find of much value. Dr. A. G. Ellis is the secretary. The library and reading rooms are located at 105 Friedrichstrasse, Ruthacker's Book Store. The meetings are held every Saturday evening at 7.30 at the Restaurant Heidelberg.

The *Texas Medical News* has moved its business office from Austin to Dallas, its new address being P. O. Box 207. The *News* is planning to make many improvements and to increase its patronage during the year 1908.

